# COVID-19 prevention behaviors, trust, and intent to vaccinate among youth at risk for HIV

**DOI:** 10.1371/journal.pone.0266321

**Published:** 2022-03-31

**Authors:** Joan Christodoulou, Anne E. Fehrenbacher, Elizabeth H. Shaw, Eleanor M. Vincent, Jessica L. Saleska

**Affiliations:** 1 Department of Psychology, Palo Alto University, Palo Alto, CA, United Stated of America; 2 Department of Psychiatry & Biobehavioral Sciences, Semel Institute, University of California, Los Angeles, CA, United States of America; Post Graduate Institute of Medical Education and Research (PGIMER), INDIA

## Abstract

The current study examines COVID-19 prevention behaviors and vaccine intentions among 83 youth at high risk for HIV. Most youth self-identified as Latinx (52%), cisgender men (84%), and homosexual (66%). Youth self-reported COVID-19 prevention behaviors and intentions to vaccinate. Participants reported wearing face masks, washing hands, and staying six feet apart, but fewer reported leaving home only for essential needs. About one-third reported that they would not get a vaccine, and lack of trust in their doctors and the government were significantly associated with non-intention. To improve efforts towards herd immunity, interventions to improve health messaging from trusted sources for at-risk youth may be necessary to achieve higher vaccine uptake.

## Introduction

Youth at high risk for HIV may also be at increased risk for a SARS-CoV-2 infection, as at-risk youth identify with sexual, gender, and racial/ethnic minority status and are more likely to have lifetime histories of homelessness, incarceration, and high-risk sexual behaviors and substance use [1,2]. Youth of color are particularly susceptible to infection, as they are more likely to experience overcrowding in households and unstable employment [3]. In addition, youth often experience occupational factors(e.g., working in essential industries), living situations in which social distancing is difficult to maintain (e.g., apartments with roommates), and frequent gatherings with individuals outside of their household (e.g., in school or socializing; [4]). Factors commonly associated with being at risk for HIV, sexual, gender, racial/ethnic minority status, living conditions and unstable family situations, as well as risky sexual and substance use behaviors further elevated the already increased risk for SARS-CoV-2 infection [1,2]. Furthermore, people living with HIV are at increased risk of severe disease and death due to COVID-19 compared to other groups, so people at high risk for or living with HIV must be prioritized in national COVID-19 vaccination policies [5]. Three COVID-19 vaccinations (Moderna, Pfizer, and Johnson & Johnson) have been authorized for emergency use by the U.S. Food and Drug Administration (FDA) [6,7] but as of January 2022, about 21–34% of American adults reported refusal to ever get vaccinated [8]. An estimated 70 to 90% of the population will need to gain immunity for herd immunity to provide indirect protection to those who are not immune [6,9]. Increasing vaccination rates is particularly important due to the new Delta and Omicron variants which have been found to be highly contagious. The Delta and Omicron variants have significantly increased COVID-19 cases among the younger population, especially due to the return to schools and colleges in 2021 [10]. Understanding the vaccination intentions, and determinants of these intentions, among youth most at risk for infection will be crucial for achieving herd immunity and for society to return to normal [11–13].

Minimal research has explored the extent and determinants of COVID-19 vaccine intent among youth [14], despite similar seroprevalence among adolescents versus adults [15–17]. A global survey across 18 countries found that potential COVID-19 vaccine acceptability was lower among 18-24-year-old individuals compared to older age groups, men compared to women, and among those with low versus high levels of trust in the government [18]. Lack of trust in health information from governmental authorities and medical providers can contribute to vaccine hesitancy. The COVID-19 pandemic has led to an increase of health misinformation, inequality-driven mistrust [19,20] and skepticism of vaccine development, especially among young men of color [11,21]. The current cross-sectional study examines COVID-19 prevention behaviors, intentions to get the vaccine, and trust in health information from a range of sources in a sample of primarily Latinx and African American youth in Los Angeles who are at high risk for HIV and may also be at higher risk of SARS-CoV-2 infection.

## Methods

### Ethics statement

The Institutional Review Board of University of California Los Angeles approved this study (IRB#16-001858-AM-00007).

### Participants

Youth aged 18–27 at-risk for HIV in Los Angeles enrolled in a pilot study examining the preferences and acceptability of Pre-Exposure Prophylaxis (PrEP) since February 2020 were recruited to participate in the current study about COVID-related behaviors from March to April 2020. Informed written consent was obtained electronically using Qualtrics and Zoom. The youth were also concurrently enrolled in the ATN149 CARES longitudinal trial investigating text-messaging, online peer support, and coaching strategies to optimize the HIV prevention continuum among seronegative youth in Los Angeles and New Orleans [22]. At-risk for HIV is based on multiple factors including sexual, gender, and racial/ethnic minority status; lifetime histories of homelessness and incarceration; and recent (last 12 months) high-risk sexual and substance use behaviors, factors which can increase risk for both HIV and SARS-CoV-2 infection.

### Measures

All participants self-reported demographic information including race/ethnicity, gender identity, sexual identity, health insurance, employment status (see Table 1) and the following assessments using an online survey distributed through Qualtrics.

**Table 1:** Demographic and behavioral characteristics of participants (N = 83).

Demographic information		*N*	%
*Race/Ethnicity*	Black/African America	13	15.7
	Latinx	43	51.8
	White	16	19.3
	Asian or Pacific Islander	8	9.6
	Other^a^	3	3.6
*Gender identity*	Cisgender man	70	84.3
	Cisgender woman	1	1.2
	Gender non-conforming, genderqueer, non-binary, two-spirit	10	12.1
	Transgender man	2	2.4
*Sexual identity*	Homosexual	55	66.3
	Heterosexual	4	4.8
	Other LGBTQ+^b^	24	28.9
*Health insurance status*	Insured^c^	61	73.5
	Uninsured	10	12.0
	Missing/unknown	12	14.5
*Employment status*	Employed (full or part-time)	47	56.6
	Unemployed, student	10	12.1
	Unemployed	26	31.3

^a^ Includes American Indian, Alaskan Native, Other (non-Latinx) racial/ethnic identities.

^b^ Includes bisexual, queer, same gender loving, downe, pansexual, asexual, and questioning sexual orientations.

^c^ Includes public, private, student, and military-based insurance.

#### COVID-19 vaccine intentions

Youth reported if they planned to get a COVID-19 vaccine when available (Yes/No) and their reasons (open-ended).

#### COVID-19 prevention behaviors

Youth reported the frequency they engaged in the following behaviors: wearing a face mask in public spaces, washing hands, staying six feet apart from others, only leaving home for essential activities, and limiting contact to those in their households (see Table 2).

**Table 2:** Frequency of prevention behaviors in past 2 months (N = 83).

Prevention behaviors		*N*	%
*Wearing a face mask in public*	Never or almost never	2	2.4
	Occasionally or sometimes	3	3.6
	Almost every time or every time	75	90.4
	Missing	3	3.6
*Washing hands*	Never or almost never	1	1.2
	Occasionally or sometimes	7	8.4
	Almost every time or every time	70	84.3
	Missing	5	6.0
*Only leaving home for essential activities*	Never or almost never	8	9.6
	Occasionally or sometimes	24	28.9
	Almost every time or every time	47	56.6
	Missing	4	4.8
*Staying 6 feet apart from others*	Never or almost never	4	4.8
	Occasionally or sometimes	16	19.3
	Almost every time or every time	58	69.9
	Missing	5	6.0
*Employment status*	Never or almost never	13	15.7
	Occasionally or sometimes	20	24.1
	Almost every time or every time	45	54.2
	Missing	5	6.0

#### Trust

Youth reported their level of trust in their personal doctor and the government regarding vaccines, and in COVID-19 related information from the Centers for Disease Control and Prevention (CDC), World Health Organization (WHO), and FDA (see Table 3).

**Table 3:** Trust in government and medical authorities, total and by intentions to get a COVID-19 vaccine (N = 83).

Trust		Total (N = 83)		Intention (n = 54)		No intention (n = 29)	
		*N*	*%*	*N*	*%*	*N*	*%*
*How much do you trust the government when it comes to vaccines*?*	Never to sometimes	58	69.9	30	55.6	28	96.6
	Nearly always to always	24	28.9	23	42.6	1	3.4
*How much do you trust your own personal doctor when it comes to vaccines*?	Never to sometimes	35	42.2	15	27.8	20	69.0
	Nearly always to always	48	57.8	39	72.2	9	31.0
*How much do you trust the information about COVID-19 that comes from CDC*?	Never to sometimes	37	44.6	18	33.3	19	65.5
	Nearly always to always	46	55.4	36	66.7	10	34.5
*How much do you trust the information about COVID-19 that comes from WHO*?	Never to sometimes	35	42.2	17	31.5	18	62.1
	Nearly always to always	48	57.8	37	68.5	11	37.9
*How much do you trust the information about COVID-19 that comes from U*.*S*. *FDA*?	Never to sometimes	48	57.8	27	50.0	21	72.4
	Nearly always to always	35	42.2	27	50.0	8	27.6

*One participant chose not to answer this question.

### Statistical analysis

We used Pearson *χ*2 tests to compare demographic and behavioral characteristics, COVID-19 prevention behaviors, and reported levels of trust in government and medical authorities by intentions to get a COVID-19 vaccine (No intention/ Intention). We also conducted logistic regression models assessing the likelihood of vaccination intention associated with levels of trust in government and medical authorities.

## Results

### Demographic and behavioral characteristics

Youth were on average 23 years old (*SD* = 2.3), and the majority self-identified as Latinx (52%), cisgender men (84%), and homosexual (66%). Most participants had health insurance (74%) and were employed part- or full-time (57%) (Table 1). None of these characteristics were significantly associated with vaccination intention.

### COVID-19 prevention behaviors

In the previous two months most (54% - 90%) youth engaged in COVID-19 prevention behaviors frequently (i.e., ‘Almost every time’ to ‘Every time’). The most commonly reported prevention behaviors were wearing face masks (90%), washing hands (84%), and staying six feet apart from others (70%). Lower proportions reported leaving the home only for essential activities (57%) and limiting contacts to those inside one’s household (54%) (Table 2). The associations between COVID-19 prevention behaviors reported and intention to vaccinate were not statistically significant in either the bivariate or multivariable analyses.

### Vaccination intention and trust

One third (35%) of youth reported that they do not intend to get a COVID-19 vaccine when it is available. Reasons for not intending to get vaccinated included mistrust in vaccines, generally (e.g., “I don’t trust vaccines”), desire to wait and see what happens to others who receive a COVID-19 vaccine, specifically (e.g., “I would have to see the results of people getting the vaccine”), and concerns regarding safety and side effects (e.g., “We don’t know the long-term effects it may have”).

In the bivariate comparisons, a substantial proportion of participants reported that they do not frequently (i.e., ‘Never’, ‘Almost never’, or ‘Sometimes’) trust the government (70%) or their personal doctor (42%) concerning vaccines, and they do not frequently trust the CDC (45%), WHO (42%), and FDA (58%) concerning COVID-19 related information. Infrequent trust in the government and in one’s personal doctor concerning vaccines were associated with non-intention to get a COVID-19 vaccine. Similarly, infrequent trust in the CDC, WHO, and FDA regarding COVID-19 related information was associated with non-intention to get a COVID-19 vaccine (Table 3).

In the logistic regression analyses, youth who reported a higher levels of trust in the government (*OR* = 3.51, *95%C*.*I*. = 1.97, 6.25, *p* < .001), their doctor (*OR* = 2.84, *95%C*.*I*. = 1.76, 4.60, *p* < .001), the CDC (*OR* = 2.11, *95%C*.*I*. = 1.39, 3.19, *p* < .001), the WHO (*OR* = 1.98, *95%C*.*I*. = 1.29, 3.04, *p* = .002), and the FDA (*OR* = 1.89, *95%C*.*I*. = 1.24, 2.90, *p* = .003) were significantly more likely to intend to receive the vaccine.

## Discussion

This cross-sectional study investigated the intention to receive a COVID-19 vaccine among youth at risk for HIV in Los Angeles County. In the current sample, 35% of youth reported that they do not intend to get a COVID-19 vaccine. Current resistance in receiving the vaccination among adults [6,9,12] poses a risk to the nation failing to reach herd immunity, making the vaccination of youth even more important [23] In this study, youth were identified as at-risk for HIV based on behavioral and structural factors which have also been shown to increase risk for SARS-CoV-2 infection (e.g., homelessness, incarceration, multiple sexual partners, frequent contacts outside of the home, etc; [24]. The rate of non-intention for vaccination among these at-risk youth is even higher than adults currently in the U.S. [25]. As COVID-19 vaccines are now available and accessible to children as young as 5 years old, targeting groups at high risk for infection will continue to be an important priority to achieve herd immunity [13].

Lack of trust in health information from personal doctors, government, and public health agencies, were all significant determinants of non-intention to receive a COVID-19 vaccine among youth in our sample. Most respondents reported trust in their personal doctor but not in the government regarding vaccines. This is consistent with recent reports of trust in scientific research and likeliness to vaccinate in France [26] and Italy [27]. Previous research on young adults has demonstrated an association between low trust in government and non-compliance with COVID-19 prevention guidelines, such as social distancing [28]. The findings from the current study further demonstrate the importance of trust on COVID-19 vaccination intentions.

Approximately half of participants in our sample reported that they do not frequently limit their contact to those within their household or leave home only for essential activities. With fewer COVID-19 restrictions now in force than in 2020 when our study was conducted, it is likely that youth are now engaging in these preventive behaviors even less frequently [29] Although COVID-19 incidence was highest among older adults in the United States early in the pandemic, by summer 2020, youth aged 18–29 years old accounted for a higher proportion of new cases than any other age group [3,30]. As of January 2022, approximately 11.3 million COVID-19 cases have been reported among youth 18–29 years old–more than any other age group nationally [31] and COVID-19-related hospitalizations among youth have now reached all-time highs during the Omicron surge [32]. Despite these alarming case rates, less than 60% of youth in the U.S. under the age of 25 years old are fully vaccinated [33].

Our findings are consistent with a recent CDC study, which identified healthcare providers as one of the most trusted sources for information on vaccinations [34]. The study noted that information on the safety and efficacy of COVID-19 vaccines was the most commonly reported factor that would increase vaccination intentions among adolescents. Future research should explore optimal diffusion strategies for disseminating information on COVID-19 vaccines to youth with the goal to maximize reach while still being sufficiently local and personal to maintain trust. Strategies to communicate with the public about the benefits, safety, and efficacy of COVID-19 vaccines should explore a range of modalities used by youth including social media and online peer support networks, such as those employed by the HIV prevention parent study for this pilot [22]. The success of HIV prevention programs utilizing peer health educators (e.g., PrEP Champions, TRIUMPH, HIVE, Advocates for Youth, etc. [35–37] offer a useful model that can be adapted for COVID-19 vaccination campaigns with youth. For example, the Public Health Institute’s FACES for the Future program trained 300 students in its health professional training initiative to build a COVID-19 peer educator workforce [38]. Leveraging their existing Public Health Youth Corps, 300 youth in California, New Mexico, Colorado, and Michigan primarily from low-income communities of color worked in 86 zip codes and 21 languages to train their local communities about COVID-19 prevention behaviors and vaccines [39].

Our findings also point toward important policy implications, particularly related to the role of trust in COVID-19 vaccination mandates targeting youth in schools and colleges, as well as new policies that could allow minors to be vaccinated without parental consent. California recently became the first state in the nation to require all youth attending school in-person to be fully vaccinated against COVID-19 [40]. However, several school districts have delayed implementing the mandate due to court challenges brought by parental rights groups [41]. Without sufficient opportunities for communities to voice their concerns regarding vaccination mandates, trust may further erode between families, local school districts, and state governments. For mandates to be successful, clear and accurate communication about vaccines must be coupled with resources to promote uptake and buy-in among both youth and their parents while minimizing the potential punitive consequences of non-compliance which could contribute to barriers to educational attainment [42]

Policies that allow youth to be vaccinated without parental involvement could also increase COVID-19 vaccine uptake among youth with parents who are hesitant to vaccinate their children. Currently, youth under the age of 18 are not legally able to get vaccinated without parental consent in most states [43,44]. For example, California Senate Bill 866 would permit children at least 12 years old to be vaccinated, including against COVID-19, without parental knowledge or permission [45]. This bill draws on existing state laws that allow minors to make sexual and reproductive health decisions without parental consent, policies which have been crucial for ensuring safe, affirming, and timely care for youth at risk or living with HIV [43].

### Limitations

The current study was limited by a small sample size, the timing of data collection, potential self-reporting bias, issues with open-ended questions, and was restricted to only online procedures as it was conducted during the COVID-19 pandemic. This study recruited from a small pilot of youth at high risk for HIV with the aim to run during the first months of the pandemic before COVID-19 vaccines were available. As a result, we were not able to evaluate if vaccination intentions influenced actual COVID-19 vaccination behaviors, or whether preventive behaviors and trust in government and health agencies changed over time as the pandemic progressed. Finally, we could not assess the impact of parental vaccine hesitancy on youth vaccination intentions. Despite these limitations, our findings highlight important opportunities for intervention, such as strengthening relationships between youth and their personal doctors, who are more trusted among youth and likely to have a stronger influence on vaccination decisions than more distal government institutions.

To address low COVID-19 vaccine uptake among at-risk youth, public information campaigns are needed to restore trust in messaging from health and government authorities and strengthen relationships between youth and their doctors. At risk adolescents must be a priority population for COVID-19 vaccination as a protective measure for themselves and for the population at large to achieve herd immunity [46].

## Conclusion

Youth at high risk for HIV are also disproportionately exposed to SARS-CoV-2 and should be prioritized for COVID-19 vaccination. Previous research has found that health care providers are the most trusted source for vaccination information among youth, and provider recommendations are a key determinant of vaccine uptake [47,48]. In this study, twice the proportion of youth reported trusting their personal doctor compared to the government regarding vaccines. As such, interventions to build and sustain trust with personal doctors and distribute health messages through these trusted sources might offer a more proximal and feasible goal to achieve high COVID-19 vaccine uptake among at-risk youth. Peer education programs and policies that promote the autonomy of youth to make their own health decisions also offer promise for improving COVID-19 vaccination rates among at-risk youth.
